# Cost drivers in the pharmacological treatment of interstitial lung disease

**DOI:** 10.1186/s12931-021-01807-8

**Published:** 2021-08-03

**Authors:** Phillen Nozibuyiso Maqhuzu, Michael Kreuter, Thomas Bahmer, Nicolas Kahn, Martin Claussen, Rolf Holle, Larissa Schwarzkopf

**Affiliations:** 1 Helmholtz Zentrum München – German Research Center for Environmental Health (GmbH), Institute of Health Economics and Health Care Management, Comprehensive Pneumology Center Munich (CPC-M), Member of the German Center for Lung Research (DZL), Ingolstaedter Landstrasse 1, 85764 Neuherberg, Germany; 2 Center for Interstitial and Rare Lung Diseases, Pneumology, Thoraxklinik, University of Heidelberg, and German Center for Lung Research (DZL), Röntgenstr. 1, 69126 Heidelberg, Germany; 3 LungenClinic Grosshansdorf GmbH Pneumology, Member of the German Center for Lung Research (DZL), Wöhrendamm 80, 22927 Großhansdorf, Germany; 4 University Hospital Schleswig-Holstein Campus Kiel, Internal Medicine I, Member of the German Center for Lung Research (DZL), Arnold-Heller-Str. 3 /Haus 41a, 24105 Kiel, Germany; 5 Institute for Medical Information Processing, Biometry, and Epidemiology, Marchioninistr. 15, 81377 Munich, Germany; 6 Institut Fuer Therapieforschung (IFT), Leopoldstr. 175, 80804 Munich, Germany

**Keywords:** Diffuse parenchymal lung disease, Healthcare expenditure, Direct costs, ILD management, Healthcare spending

## Abstract

**Introduction:**

Treatments of interstitial lung diseases (ILDs) mainly focus on disease stabilization and relief of symptoms by managing inflammation or suppressing fibrosis by (in part costly) drugs. To highlight economic burden of drug treatment in different ILD-subtypes we assessed cost trends and therewith-associated drivers.

**Methods:**

Using data from the German, observational HILDA study we estimated adjusted mean medication costs over 36-month intervals using one- and two-part Generalized Estimating Equation (GEE) regression models with a gamma distribution and log link. Next, we determined factors associated with costs.

**Results:**

In Idiopathic pulmonary fibrosis (IPF) mean per capita medication costs increased from €1442 before to €11,000€ at the end of study. In non-IPF subtypes, the increase took place at much lower level. Mean per capita ILD-specific medication costs at the end of the study ranged between €487 (other ILD) and €9142 (IPF). At baseline, higher FVC %predicted values were associated with lower medication costs in IPF (−9%) and sarcoidosis (−1%). During follow up higher comorbidity burden escalated costs in progressive fibrosing ILD (PF-ILD) (+52%), sarcoidosis (+60%) and other ILDs (+24%). The effect of disease duration was not uniform, with cost savings in PF-ILD (−8%) and sarcoidosis (−6%), but increased spending in IPF (+11%).

**Conclusion:**

Pharmacological management of ILD, in particular of IPF imposes a substantial economic burden on the healthcare system. Strategies to reduce comorbidity burden and early treatment may reduce the impact of ILDs on the healthcare system.

**Supplementary Information:**

The online version contains supplementary material available at 10.1186/s12931-021-01807-8.

## Introduction

The rare group of interstitial Lung Diseases (ILDs) comprises of over 200 subtypes that are heterogeneous regarding etiology, patterns and prognosis, but have similar pathophysiological pathways regarding inflammation and/or fibrosis of the lung parenchyma [1]. ILDs are classified as idiopathic, granulomatous (e.g. sarcoidosis) or associated with known causes [2, 3].

Many ILDs are triggered by environmental, occupational, or medication-related exposures [4] such as e.g. hypersensitivity pneumonitis (HP) [5], and drug-induced ILD [6]. Other ILDs are pulmonary manifestations from systemic autoimmune diseases, [7] such as rheumatic arthritis and connective tissue disease (R-CTD) [4]. Idiopathic ILDs, also referred to as idiopathic interstitial pneumonias (IIPs)—which include idiopathic pulmonary fibrosis (IPF) as their most prominent form—represent an important subset which is associated with a substantial loss of quality of life [8, 9] and with a detrimental survival [10].

Within the various non-IPF ILDs, a proportion of patients can develop a progressive, fibrosing phenotype. This phenotype is characterized by declining lung function and high mortality [11, 12]. In contrast to IPF however, progressive fibrosing ILDs (PF-ILD) are often accompanied by inflammation. Around 20–30% of ILDs may develop into a PF-ILD [12]. ILDs that are associated with this phenotype include sarcoidosis, non-specific interstitial pneumonia (NSIP), CTD-ILD, HP and unclassifiable ILD [11].

ILD treatments mainly focus on disease stabilization and on relief of symptoms by managing inflammation and/or suppressing fibrosis [4]. In this regard, most evidence—particularly on pharmaceutical interventions—is based on treatment of IPF patients with two licensed anti-fibrotic treatments, whereas in non-IPF ILDs evidence-based treatment approaches and thus guidelines are mainly lacking.

Given their mostly chronic, often progressive course combined with the often non-standardized treatment regimens, ILDs might be associated with high healthcare utilization and a significant strain and burden on health services, and healthcare systems [13–15]. Most evidence corresponding to the economic burden of ILDs is however restricted to distinct service providers (e.g. hospital costs) [16], focused on pre-selected ILD subtypes [17, 18] or took a cross-sectional perspective on costs of care [14]. A comprehensive assessment on costs associated with drug treatment that accounts for potentially different trends in various ILDs is lacking so far. In addition, as these chronic diseases are often accompanied by diverse comorbidities [19] an in-depth study to determine the overall cost of drug treatment in ILD patients is crucial.

To close this knowledge gap, our analysis of the HILDA-cohort study portrays the course of medication costs over an 18-months interval and delineates essential cost drivers in pharmacological treatment. The anticipated results are expected to aid German policy makers in planning and allocating healthcare services.

## Methods

### Study design and population

The German longitudinal HILDA (Health Care in ILD Outpatient Visitors) cohort is a prospective, observational study [9, 20]. Recruitment took place between November 2016 and April 2017 with patient-individual follow-up intervals after 6 months (t1) and 12 months (t2). The study collected information on pharmaceutical treatment retrospectively at each follow up visit. Pharmaceutical data at baseline therefore corresponds to the time before the study began, t1 corresponds to the first 6 months after the recruitment and t2 corresponds to the interval from six to 12 months after recruitment.

Inclusion criteria were an ILD-diagnosis confirmed by the multidisciplinary team board meeting of the recruiting center, a minimum age of 18 years, proficiency in the German language and the provision of written informed consent. In total, 271 patients enrolled into the study.

### Medication costs

We calculated drug costs based on information on the Pharmaceutical Central Number of the distinct drug and dosage of drug intake per day during the study period by applying drug-specific prices stemming from the medication database of Scientific Institute of the AOK Statutory Health Insurance funds (WIdO 2016). Apart from nutritional supplements, we considered all pharmaceuticals taken by the patient. Each drug was classified as an ILD-related medication (immunosuppressants, steroids, pirfenidone, nintedanib) or other drug. Subsequently, we calculated medication costs for the three study intervals, 6 months before baseline (t0), baseline to 6 months after baseline (t1) and 6 to 12 months after baseline (t2).

### Covariates

We considered the baseline covariates age (in years), sex, duration of disease (in years), smoking status (current or former and never-smoker), study center, comorbidity burden and physician-reported information on forced vital capacity (FVC) % predicted. In addition, we considered four different ILD subtypes: (1) Sarcoidosis, (2) IPF, (3) PF-ILD (determined by a decrease of at least 10% in either FVC %predicted or DLCO %predicted values after 12 months [12]), as well as (4) other ILD subtypes. Other ILD subtypes comprised of idiopathic interstitial pneumonias, hypersensitivity pneumonitis, rheumatic and connective tissue diseases with pulmonary involvement, drug-related ILD, combined pulmonary fibrosis and emphysema, non-classifiable ILD and other forms. A detailed list of this subgroup is available in Additional file 1: Appendix Table S1.

The pre-determined list of comorbidities included pulmonary hypertension, arterial hypertension, coronary heart disease, congestive heart failure, other cardiovascular disease, diabetes mellitus, emphysema/COPD, lung cancer, depression, gastroesophageal reflux disease, renal failure, obstructive sleep apnea, thromboembolism, and malignant tumors excluding lung cancer, which were identified to be of either epidemiological or clinical relevance in ILDs [14]. Physicians had the possibility to include three other comorbidities not present in the list and the highest possible number of comorbidities was hence 17. As the number of patients with each distinct comorbid condition was small, we refrained from a separate analysis of the several conditions. To reflect comorbidity burden we instead calculated a summative index from the documented comorbid conditions which hat a possible range from 0 to 17. A comprehensive list of comorbidities and their frequencies in the sample is illustrated in Additional file 1: Appendix Table S5.

We disregarded information on diffusing capacity of carbon monoxide (DLCO) % predicted in our analyses because this led to multicollinearity problems [12].

### Statistical analysis

We disregarded participants with missing information on any variable of interest, which however only applied to disease duration (N = 12) and comorbidity sum score (N = 2). Furthermore, analyses were restricted to complete cases at the distinct assessment points. The respective samples comprised of 257 patients at t0, 229 patients at t1 and 204 patients at t2. We stratified all analyses by ILD subtype and clustered by center to account for correlation between patients from the same treatment center.

To identify structural differences between the distinct ILD subtypes, we compared baseline characteristics by Pearson’s chi-square tests (categorical variables), and Kruskal Wallis tests (continuous variables). Next, we determined the proportion of patients receiving either immunosuppressant medication, steroids or established treatment for IPF at each assessment point and assessed unadjusted mean medication costs.

The outcome of interest was the cost of all medications in ILD patients. The primary analysis estimated covariate-adjusted mean medication costs throughout the study period by ILD subtype. To address intra-subject correlation in context of repeated measures, we applied Generalized Estimating Equation (GEE) regression models with first order auto-regression [21]. To account for the right-skewed distribution of cost data, we assumed a gamma-distribution with log-link in our GEEs. As more than 10% of patients incurred zero costs, we performed two-part GEEs. Two-part models consist of a logistic regression model as part 1 and a gamma model as part 2. The logistic regression model predicts the probability of positive costs, while the gamma model estimates the costs for the subsample with positive costs. The probabilities from part 1 are multiplied by the calculated costs per user from part 2 to determine adjusted per capita costs [22, 23].

In our secondary analyses, we first assessed factors influencing baseline costs using simple gamma models, and subsequently we estimated the factors associated with costs during the study period, using gamma-distributed GEEs. We interpreted the exponentials of the regression coefficients in each of these two analyses as surcharge factors. We adjusted for the same covariates investigated at baseline and additionally accounted for the time point in the GEE models.

As a sensitivity analysis, we re-ran all analyses for those patients who participated at all three assessment points (study completers).

All statistical analyses were performed using the SAS software package (SAS Institute Inc., Cary, NC, USA, version 9.4) and we considered a p-value less than 0.05 statistically significant.

## Results

### Comparison of patient characteristics in different ILD subgroups at baseline

Table 1 describes the sociodemographic characteristics of the study sample stratified by ILD subtype. Across all ILD subtypes, most patients were male and never smokers. The ILD subtypes differed in terms of disease duration, age and comorbidity burden, with the difference being most pronounced between IPF and sarcoidosis. FVC % and DLCO% predicted differed between the distinct subtypes (p = 0.0016, p < 0.001, respectively), with PF-ILD-patients presenting the highest values at baseline. Sarcoidosis patients had longest disease duration (8.1 ± 10.1 years), were the youngest (52.0 ± 12.1) and presented with the lowest comorbidity burden (2.4 ± 1.3), whereas IPF patients had the shortest disease duration (2.4 ± 1.9 years), were the oldest (71.3 ± 6.5) and presented with the highest comorbidity burden (3.8 ± 1.7). Regarding these baseline characteristics, patients with PF-ILDs were similar to sarcoidosis patients (Table 1).

**Table 1:** Differences in patient characteristics stratified by ILD subtypes

	IPF N = 72	PF-ILD (N = 32)	Sarcoidosis N = 45	Other ILD N = 122	p-value
Mean FVC % at t0	72.9 (21.3)	83.7 (21.3)	74.6 (16.9)	67.8 (20.2)	0.0016*
Mean DLCO % at t0	38.9 (12.0)	57.3 (19.2)	55.7 (18.0)	39.0 (13.1)	0.0010*
Mean age, years (SD)	71.3 (6.5)	57.9 (13.3)	52.0 (12.1)	62.2 (12.4)	< 0.0001*
disease duration, years (SD)	2.4 (1.9)	5.4 (6.3)	8.1 (10.1)	3.1 (4.6)	< 0.0001*
Mean number of comorbidities (SD)	3.8 (1.7)	2.5 (1.3)	2.4 (1.3)	2.8 (1.5)	< 0.0001*
Male (%)	57 (79.2)	23 (71.2)	33 (73.3)	66 (54.1)	0.0020*
Never smoker (%)	19 (26.4)	16 (50.0)	19 (42.2)	46 (37.7)	0.0955

^*^p-value < 0.05

### Proportion of patients receiving different therapies

The majority of non-IPF patients were administered steroids for the treatment of ILD, 52.5% (N = 135/257), 57.6% (N = 132/229) and 50.0% (N = 102/204) at t0, t1 and t2 respectively. Immunosuppressant medication was used by 33.1% (N = 85) at t0, 28.4% at t1 (N = 65) and 29.9% (N = 61) at t2. A few patients received the combination therapy of steroids and immunosuppressant medication 19.5% (N = 50), 21.4% (N = 49) and 22.1% (N = 45) during the respective time points. At t0 and t1, respectively, about one in five IPF patients received either pirfenidone or nintedanib (t0: N = 15; 20.8%|t1: N = 12; 19.0%), whereas this treatment quota at t2 was 100% (N = 55). Furthermore, a small subset of patients who started on steroid therapy switched to either pirfenidone or nintedanib during the 6-month interval after receiving an official IPF diagnosis, (N = 3 (1.2%), N = 7 (3.1%) and N = 9 (4.4%) at t0, t1 and t2 respectively).

### Unadjusted mean medication costs in different ILD subtypes over time

At t0, unadjusted medication costs ranged between €132 (Fibrosing ILD) and €332 (IPF). For sarcoidosis and IPF, costs had multiplied by t1 already and for all subtypes, there was a steep increase of costs between t1 and t2.

### Adjusted mean medication costs in different ILD subtypes over time

**Fig. 1:**
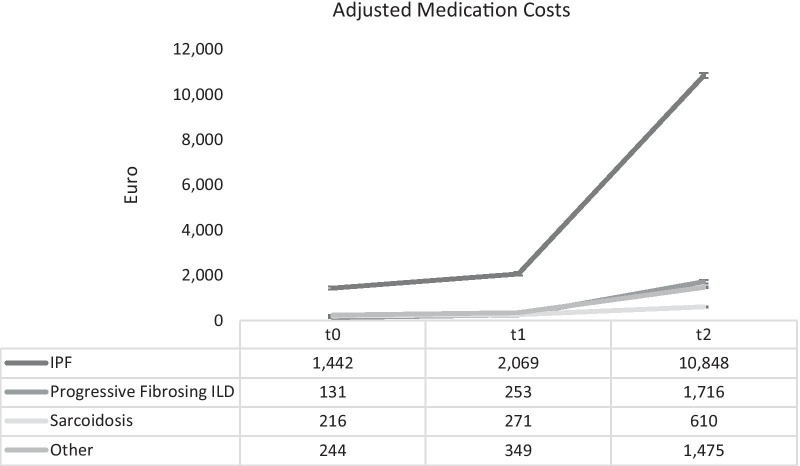
Adjusted mean overall medication costs from baseline to 12 months across different ILD subtypes

**Fig. 2:**
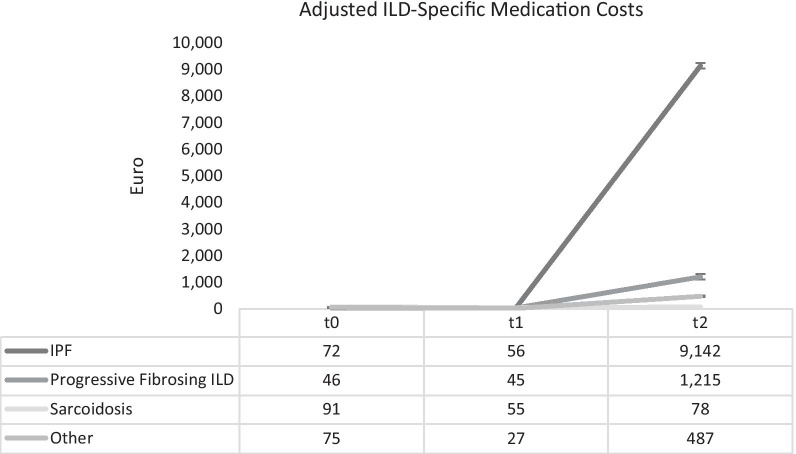
Adjusted ILD-specific mean medication costs from baseline to 12 months across different ILD subtypes

Figures 1 and 2 display the adjusted medication costs for all treatments and ILD-specific treatments respectively. At t0, before study began medication costs for the distinct subtypes varied between about €216 (sarcoidosis) and about €1442 (IPF). During the study, costs for IPF patients increased from ~ €2000 to ~ €11,000. In the non-IPF subtypes, there was also a substantial increase, taking place at much lower level. Spending on ILD-specific medication ranged from €46 (PF-ILD) to €91 (sarcoidosis) before at t0. This amount decreased slightly during the first 6 months of the study but increased again sharply after t1 in all subtypes except sarcoidosis. The ILD-specific medication costs at the end of the study ranged from €487 (other ILD) to €9,142 (IPF).

### Factors influencing medication costs at baseline and over time

Table 2 illustrates drivers of baseline costs. Across all ILD subtypes increase of comorbidity burden was associated with a significant increase in medication costs that ranged between + 23% (sarcoidosis) and + 89% (other ILDs). Disease duration was associated with significantly lower expenditures in non-IPF patients. Older age had a cost driving effect in IPF (+ 10%) and PF-ILD (+ 4%) and other ILDs (+ 3%). Smoking was associated with a 247% increase in costs in IPF. Higher FVC % predicted values at baseline were associated with lower costs in IPF and sarcoidosis patients only, with an impact of −9% and −1% respectively.

**Table 2:** Influencing baseline factors on medical costs across ILD subtypes

	IPF	PF-ILD	Sarcoidosis	Other ILD
	surcharge factor [95% CI]	surcharge factor [95% CI]	surcharge factor [95% CI]	surcharge factor [95% CI]
Male	0.06 [0.00–0.22]	1.05 [0.77–1.34]	0.93 [0.84–1.03]	1.67 [1.59–1.76]*
Former/Current smoker	2.47 [2.40–2.55]*	0.41 [0.00–0.83]	0.91 [0.57—–1.25]	0.82 [0.54–1.11]
Age	1.10 [1.09–1.10]*	1.04 [1.03–1.06]*	1.00 [0.99–1.00]	1.03 [1.02–1.04]*
FVC % pred. At baseline	0.91 [0.90–0.92]*	0.99 [0.98–1.00]	0.99 [0.99–0.99]*	0.99 [0.99–1.00]
Disease duration	1.02 [0.89–1.15]	0.90 [0.90–0.91]*	0.95 [0.93–0.97]*	0.91 [0.91–0.91]*
Comorbidity sum score	1.59 [1.49–1.69]*	1.50 [1.41–1.59]*	1.23 [1.19–1.26]*	1.89 [1.80–1.97]*

^*^p-value < 0.05

Table 3 displays costs drivers of the development of medication costs over the study period. A high comorbidity burden was linked to higher medication costs in PF-ILD (+ 52%), sarcoidosis (+ 60%) and other ILDs (+ 24%). A longer disease duration had a cost-saving impact in PF-ILD (−8%) and sarcoidosis (−6%), and in contrast had a cost-driving effect in IPF (+ 11%). Older age had a cost-driving impact on PF-ILD while smoking had a cost-driving impact in sarcoidosis. Male gender was associated with higher costs in PF-ILD and sarcoidosis. Medication costs at t2 were significantly higher in all subtypes than at t0, while costs at t1 were significantly higher than costs at t0 in PF-ILD only.

**Table 3:** Influencing factors on longitudinal medication costs across ILD subtypes

	IPF	PF-ILD	Sarcoidosis	Other ILD
	OR [95% CI]	OR [95% CI]	OR [95% CI]	OR [95% CI]
Male	1.03 [0.65–1.41]	1.73 [1.27–2.20]*	2.56 [1.58–3.54]*	1.02 [0.64–1.40]
Former/current smoker	1.25 [0.92–1.59]	0.57 [0.13–1.00]	2.62 [1.79–3.46]*	1.00 [0.59–1.41]
Age	0.99 [0.97–1.01]	1.05 [1.03–1.07]*	1.03 [0.99–1.07]	0.99 [0.97–1.01]
FVC % pred. At baseline	1.00 [0.99–1.00]	1.00 [0.99–1.01]	1.00 [0.97–1.03]	0.99 [0.98–1.00]
Disease duration	1.11 [1.03–1.19]*	0.92 [0.88–0.96]*	0.94 [0.89–0.99]*	0.93 [0.89–0.97]*
Comorbidity sum score	1.04 [0.96–1.13]	1.52 [1.31–1.74]*	1.60 [1.23–1.97]*	1.24 [1.11–1.38]*
T1	1.24 [0.87–1.61]	2.22 [1.72–2.71]*	0.77 [0.42–1.12]	1.05 [0.67–1.42]
T2	5.95 [5.59–6.30]*	11.8 [11.3–12.3]*	2.09 [1.64–2.54]*	6.46 [6.05–6.86]*

^*^p-value < 0.05

### Sensitivity analysis

The results for the influencing factors on baseline costs and cost development for complete cases are illustrated in Additional file 1: Appendix Tables S2 and S3. For the most part, the results were similar to the main analyses. When comparing cost drivers at baseline, we found that the direction of the effect of male sex and age was reversed in IPF patients. When observing medication costs throughout the study, the direction of the effects of all covariates were the same as in the main analyses.

## Discussion

In this study, we illustrated the pharmaceutical treatment costs in patients diagnosed with various ILDs and determined the associated cost drivers in Germany. Our results showed that IPF patients had the highest medication costs of all ILD groups within the study period, which rose sharply after baseline. The reason beyond this observation is the high cost of the two approved anti-fibrotic medications for IPF, pirfenidone and nintedanib [2]. Indeed 12 months after baseline, when all IPF patients had received either pirfenidone or nintedanib, corresponding expenditures explained almost 85% of total medication costs. The steep rise in medication costs suggests that there was a rollout of these two medications until all eligible IPF patients were on these medications by the second half of the study, as shown in our results.

For non-IPF ILDs, treatment mainly relies on relatively inexpensive steroids and immunosuppressant medication, as illustrated by our results. Nevertheless, we observed a general increase in total medication costs in non-IPF patients during the study, especially in PF-ILD patients. This suggests intensified pharmaceutical treatment of comorbidities, mainly in the first six months of the study period, as depicted by the corresponding reduction of ILD-specific medication costs at t1. Regardless, 12 months after baseline, ILD-related costs had increased again, indicating intensified pharmaceutical treatment of ILD in non-IPF patients. The steep increase in medication costs in PF-ILD patients was due to three patients that were administered pirfenidone or nintedanib in t2 for compassionate use. It is to note however, that at the time of the HILDA study, these medications were not yet approved for the treatment of PF-ILD in Germany as nintedanib was approved in 2020 [24]. In future, we assume that the cost of medication in this subgroup of patients will increase and mirror IPF, considering that the costs of anti-fibrotic medication can presumably not be modified except if the patency is removed and generic medication becomes available. Although we did not evaluate cost of entire healthcare resource utilization in our study, other researchers have demonstrated that both IPF and PF-ILD patients are hospitalized more often than patients without a fibrosing phenotype [18, 25, 26], and therefore incur higher costs. For this reason, we expect anti-fibrotic medication will moderate the clinical course of the disease and, if necessary, cost-intensive hospitalizations will be reduced. Therefore, the increase in medication costs should not necessarily be viewed critically.

### Cost drivers

Comorbidity burden was a cost-driving factor in all subtypes either at baseline or throughout the study period. In addition to pharmaceutical management of the index disease, a sensitive pharmaceutical management of comorbidities is required in treatment of ILD. In IPF, a study found that comorbidities influence the clinical course of the disease and survival [27]. Accordingly, a systematic review on cost triggers in IPF identified comorbidities as a substantial factor in the increasing cost of IPF management [15] and similar findings have also been established in sarcoidosis [14].

Lower FVC % predicted values were a determinant for higher medication costs in IPF and sarcoidosis. Studies have found that declining values of FVC % predicted are linked to disease progression [28]. Patients with worsening symptoms of the disease may require higher doses or higher frequencies of pharmaceutical treatment. Moreover, sarcoidosis patients with normal lung function values do not require pharmaceutical treatment [7, 29] and this explains the cost-saving effect of higher lung function values.

In our study, a longer disease duration was associated with higher medication costs in IPF but lower medication costs in non-IPF subtypes. With anti-fibrotic treatment one only achieves slowing down the inevitable lung function decline in IPF [30, 31]. Thus, most patients need continuous medication over the entire course of their disease to alleviate symptoms and reduce lung deterioration [32]. In contrast in PF-ILDs it the rapid lung function decline itself that renders drug treatment necessary. Here anti-fibrotic treatment represents a timely-defined intervention to stabilize further lung and to avoid its further deterioration [33]. After this goal has been achieved medication can in some cases be dispensable. In the case of sarcoidosis, this disease may be acute or chronic. Some patients with acute sarcoidosis may recover spontaneously [34]. Chronic sarcoidosis, however, has a subtle onset and is slowly progressing. Thus, in many patients with chronic sarcoidosis, treatment is not required [7, 29]. However, as patients within the HILDA registry were recruited in expert centers, a significant higher treatment indication has to be expected.

Age was a cost-driver in both IPF and PF-ILD. Age has been identified as a risk factor for ILD progression [35]. Furthermore, older age is particularly known in to increase the likelihood of progression and mortality in progressive fibrosing phenotypes [12].

Male sex was associated with higher costs in PF-ILD and sarcoidosis. Risk prediction in ILD can be determined by the Gender, Age and Physiology (GAP) index model, which places men at a higher risk of mortality than women [35]. Increased mortality indicates worse outcomes for men than women, which would require more pharmaceutical treatment and therefore incur higher costs.

We demonstrated that current or former smokers had higher costs than never-smokers in IPF at baseline and in sarcoidosis patients during the study. Smoking is linked to increased inflammation in the lung, which may lead to worse outcomes [36]. Over time, however, smoking status had no cost-driving effect in IPF patients. This could be due to the mechanism of pirfenidone and nintedanib that were accessible to all IPF patients in the study. The drugs have been shown to relieve inflammation [30, 31].

### Sensitivity analysis

Factors influencing medication costs were similar in both the main and sensitivity analyses. The differences found in IPF can be explained by dropout analysis (Additional file 1: Appendix Table S4). The dropouts were significantly older and male. The remaining sample favored healthier, younger patients. Male sex therefore had a cost-saving impact in the sensitivity analysis although usually, IPF is a disease that mostly affects men, and being male is also a risk factor for the regression of disease [12].

## Strengths and limitations

So far, data on the pharmacological management of ILD in Germany are sparse. One strength of our study is that we simultaneously analyzed different ILD subtypes and compared their burden on the healthcare system as opposed to the previous study conducted in Germany that only included two subtypes of ILD and did not include clinical parameters [14]. Moreover, we were able to assess cost drivers for the most common ILD subtypes. As our study was over a longer period, we could also make concrete deductions on factors influencing medication costs.

There are several limitations to our study. An important limitation is that we did not adjust for lung transplantations, as the study did not collect the information. Several studies have found that lung transplantation and associated medications is one of the most costly component of ILD management [25]. However, generally, lung transplantations are rarely performed [37] and only a third of all lung transplantations are due to ILD [38]. Another limitation is the heterogeneous group of "other ILDs". We are not able to make concrete deductions on this subgroup due to a large variance. In addition, owing to the study design we were not able to differentiate between pulmonary sarcoidosis and systemic sarcoidosis. Hence, obtained costs reflect costs for a heterogeneous sample of sarcoidosis patients, within which those with systemic manifestations presumably incur higher costs than those with pulmonary manifestations only. Lastly, the summative consideration of comorbidity burden does not allow any conclusions to be drawn as to which combinations are particularly costly, therefore—if sample sizes allow—an analysis of individual comorbid conditions could shed further light on this important issue.

## Conclusion

The results of our study suggest that the pharmacological management of ILD, in particular that of IPF imposes a substantial economic burden on the healthcare system in Germany. Strategies to reduce comorbidity burden and lung function decline could essentially reduce the burden of all ILD on both caregivers and the healthcare system.

## Supplementary Information

**Additional file 1: Table S1.** List of ‘Other’ ILD subtypes. **Table S2.** Influencing factors on medical costs at baseline across ILD subtypes in complete cases. **Table S3.** Influencing factors on longitudinal medication costs across ILD subtypes in complete cases. **Table S4.** Characteristics of the complete and incomplete cases. **Table S5.** Frequency of comorbid conditions present at baseline by ILD subtype.

## Data Availability

The data that support the findings of this study are available on reasonable request. The data are not publicly available due to their containing information that could compromise the privacy of research participants.

## Abbreviations

CPFE: Combined pulmonary fibrosis with emphysema
CTD-ILD: Connective tissue diseases
DLCO: Diffusing capacity of carbon monoxide
FVC: Forced vital capacity
GEE: Generalized Estimating Equations
GLM: Generalized Linear Models
HILDA: Health Care in ILD Ambulance Visitors
HP: Hypersensitivity pneumonitis
HRQL: Health-related quality of life
IIPs: Idiopathic interstitial pneumonias
ILD: Interstitial lung diseases
IPF: Idiopathic pulmonary fibrosis
NSIP: Non-specific interstitial pneumonia
PF-ILD: Progressive fibrosing ILD
R-CTD: Rheumatic and connective tissue diseases with pulmonary involvement
WIdO: Scientific Institute of the AOK
