# Supplementary material for: Cost drivers in the pharmacological treatment of interstitial lung disease
Source: Respir Res. 2021 Aug 3;22:218. doi: 10.1186/s12931-021-01807-8 (PMC8335870; doi:10.1186/s12931-021-01807-8)
Supplement: Supplementary file 1 — Additional file 1: Table S1. List of ‘Other’ ILD subtypes. Table S2. Influencing factors on medical costs at baseline across ILD subtypes in complete cases. Table S3. Influencing factors on longitudinal medication costs across ILD subtypes in complete cases. Table S4. Characteristics of the complete and incomplete cases. Table S5. Frequency of comorbid conditions present at baseline by ILD subtype. [file 12931_2021_1807_MOESM1_ESM.docx]

## Appendix

Appendix Table 1: List of ‘Other’ ILD subtypes

| ILD Subtype | N (%) |
| --- | --- |
| Idiopathic Interstitial Pneumonias |  |
| - Non-specific interstitial pneumonia (NSIP) | 16 (13.1) |
| - Desquamative interstitial pneumonia (DIP) | 2 (1.6) |
| - Cryptogenetic organizing pneumonia (COP) | 2 (1.6) |
| Hypersensitivity pneumonitis (exogen allergic alveolitis) | 25 (20.5) |
| Rheumatic and connective tissue diseases with pulmonary involvement | 5 (4.1) |
| Drug-related | 3 (2.5) |
| Combined pulmonary fibrosis and emphysema (CPFE) | 5 (4.1) |
| Other Forms |  |
| - Pulmonary hymphangioleiomyomatosis | 7 (5.7) |
| - Pulmonary Langerhans cell histocytosis | 2 (1.6) |
| - Pulmonary alveolar proteinosis | 2 (1.6) |
| Others | 35 (28.7) |
| Not classifiable | 18 (14.8) |

Appendix Table 2: Influencing factors on medical costs at baseline across ILD subtypes in complete cases

|  | IPF | Fibrosing ILD | Sarcoidosis | Other |
| --- | --- | --- | --- | --- |
|  | OR [95% CI] | OR [95% CI] | OR [95% CI] | OR [95% CI] |
| Male | 0.04 [0.00 - 0.47] | 1.05 [0.77 - 1.34] | 1.47 [1.25 - 1.69]* | 1.74 [1.33 - 2.15]* |
| Former/Current smoker | 2.14 [1.85 - 2.42]* | 0.41 [0.00 - 0.83] | 0.67 [0.00 - 1.41] | 0.92 [0.85 - 0.99]* |
| Age | 0.93 [0.91 - 0.95]* | 1.04 [1.03 - 1.06]* | 0.96 [0.95 - 0.96]* | 1.02 [1.01 - 1.03]* |
| FVC % pred. At baseline | 0.93 [0.91 - 0.95]* | 0.99 [0.98 - 1.00] | 1.00 [1.00 - 1.00] | 0.99 [0.99 - 1.00] |
| Disease duration | 1.23 [1.16 - 1.30]* | 0.90 [0.90 - 0.91]* | 0.96 [0.95 - 0.96]* | 0.91 [0.87 - 0.94]* |
| Comorbidity sum score | 1.40 [1.22 - 1.57*] | 1.50 [1.41 - 1.59]* | 1.30 [1.23 - 1.37]* | 2.12 [1.82 - 2.41]* |

**p-value < 0.05*

Appendix Table 3: Influencing factors on longitudinal medication costs across ILD subtypes in complete cases

|  | IPF | Fibrosing ILD | Sarcoidosis | Other |
| --- | --- | --- | --- | --- |
|  | OR [95% CI] | OR [95% CI] | OR [95% CI] | OR [95% CI] |
| Male | 0.36 [0.22 - 0.93]* | 1.73 [1.27 - 2.20]* | 2.67 [1.87 - 3.47]* | 1.53 [1.10 - 1.95]* |
| Former/Current smoker | 1.29 [0.79 - 1.78] | 0.57 [0.13 - 1.00] | 2.61 [1.90 - 3.33]* | 1.58 [1.12 - 2.03]* |
| Age | 0.98 [0.94 - 1.01] | 1.05 [1.03 - 1.07]* | 1.03 [0.99 - 1.06] | 1.02 [1.00 - 1.04] |
| FVC % pred. At baseline | 1.00 [0.99 - 1.01] | 1.00 [0.99 - 1.01] | 0.99 [0.96 - 1.01] | 1.01 [1.00 - 1.02] |
| Disease duration | 1.09 [0.96 - 1.21] | 0.92 [0.88 - 0.96]* | 0.94 [0.90 - 0.98]* | 1.04 [1.00 - 1.08] |
| Comorbidity sum score | 0.99 [0.85 - 1.13] | 1.52 [1.31 - 1.74]* | 1.60 [1.27 - 1.93]* | 1.15 [1.01 - 1.29]* |
| T1 | 4.65 [4.09 - 5.20]* | 2.22 [1.72 - 2.71]* | 0.96 [0.58 - 1.34] | 1.56 [1.11 - 2.00]* |
| T2 | 38.41 [37.86 - 38.95]* | 11.8 [11.3 - 12.3]* | 3.51 [3.03 - 3.98]* | 1.58 [1.12 - 2.03]* |

**p-value* < 0.05

Appendix Table 4: Characteristics of the complete and incomplete cases

|  | Complete cases | Incomplete cases | p-value |
| --- | --- | --- | --- |
| N | 200 | 71 |  |
| FVC % predicted, mean (SD) | 74.1 (21.0) | 66.7 (18.6) | 0.0078* |
| DLCO % predicted, mean (SD) | 44.8 (16.1) | 41.5 (17.3) | 0.0741 |
| Mean age, years (SD) | 61.4 (12.7) | 65.2 (13.1) | 0.0132* |
| Mean time since diagnosis, years (SD) | 4.5 (6.6) | 2.6 (3.5) | 0.0035* |
| Mean number of comorbidities (SD) | 2.9 (1.6) | 3.1 (1.5) | 0.6328 |
| Male (%) | 133 (66.5) | 46 (64.8) | 0.7936 |
| Female (%) | 67 (33.5) | 25 (35.2) |  |
| ILD subtypes: |  |  | 0.0012* |
| IPF (%) | 55 (27.5) | 17 (23.9) |  |
| Fibrosing ILD | 32 (16.0) | 0 (0.0) |  |
| Sarcoidosis % | 33 (16.5) | 12 (16.9) |  |
| Other (%) | 80 (40.0) | 42 (59.2) |  |
| Smoking status: |  |  | 0.8187 |
| Current/Former (%) | 127 (63.5) | 44 (62.0) |  |
| Never smoker | 73 (36.5) | 27 (38.0) |  |

**p-value < 0.05*

Appendix Table 5: Frequency of comorbid conditions present at baseline by ILD subtype

|  | IPF  (N = 71) | PF-ILD  (N = 32) | Sarcoidosis  (N = 45) | Other ILD  (N = 122) |
| --- | --- | --- | --- | --- |
| Pulmonary Hypertension | 5 (7.0) | 1 (3.1) | 2 (4.4) | 9 (7.4) |
| Arterial hypertension | 36 (50.7) | 9 (28.1) | 20 (44.4) | 46 (37.7) |
| Coronary heart disease | 30 (42.3) | 5 (15.6) | 2 (4.4) | 16 (13.1) |
| Congestive heart failure | 3 (4.2) | 0 (0.0) | 3 (6.7) | 5 (4.1) |
| Other cardiovascular disease | 2 (2.8) | 0 (0.0) | 0 (0.0) | 3 (2.5) |
| Diabetes mellitus | 24 (33.8) | 4 (12.5) | 5 (11.1) | 27 (22.1) |
| Emphysema/COPD | 0 (0.0) | 0 (0.0) | 0 (0.0) | 0 (0.0) |
| Lung cancer | 0 (0.0) | 0 (0.0) | 0 (0.0) | 0 (0.0) |
| Depression | 6 (8.5) | 2 (6.3) | 5 (11.1) | 7 (5.7) |
| Gastroesophageal reflux disease | 8 (11.3) | 1 (3.1) | 0 (0.0) | 11 (9.0) |
| Renal failure | 4 (5.6) | 2 (6.3) | 1 (2.2) | 5 (4.1) |
| Obstructive sleep apnea | 11 (15.5) | 6 (18.8) | 2 (4.4) | 7 (5.7) |
| Thromboembolism | 1 (1.4) | 0 (0.0) | 3 (6.7) | 3 (2.5) |
| Malignant tumors excluding lung cancer | 4 (5.6) | 1 (3.1) | 1 (2.2) | 1 (0.8) |

*Data are presented as N (%)*
